# Artificial Cold Air Increases the Cardiovascular Risks in Spontaneously Hypertensive Rats

**DOI:** 10.3390/ijerph9093197

**Published:** 2012-09-04

**Authors:** Bin Luo, Shuyu Zhang, Shoucun Ma, Ji Zhou, Baojian Wang

**Affiliations:** 1 School of Applied Meteorology, Nanjing University of Information Science and Technology, 219 Ningliu Road, Nanjing 210044, China; Email: luobin07@lzu.cn (B.L.); msc_ever@163.com (S.M.); zhoujigood@163.com (J.Z.); 2 Key Laboratories of Arid Climatic Change and Reducing Disaster of Gansu Province, Lanzhou Institute of Arid Meteorology, CMA, 2070 Donggang East Road, Chengguan District, Lanzhou 730020, China; 3 Lanzhou Central Meteorological Observatory, 2070 Donggang East Road, Chengguan District, Lanzhou 730020, China; Email: baojianwang@126.com

**Keywords:** artificial cold air, SHR, atherosclerosis risk factors

## Abstract

The purpose was to investigate the effects of artificial cold air on cardiovascular risk in hypertensive subjects. An artificial cold air was simulated with hourly ambient temperature data of a real moderate cold air in China. Twenty-four male SHR rats were randomly divided into the minimum temperature (Tmin) group, the rewarming temperature (Tr) group and two concurrent control groups with six rats in each (Tmin and Tr represent two cold air time points, respectively). Tmin and Tr groups were exposed to the cold air that was stopped at Tmin and Tr, respectively. After cold air exposure, blood pressure, heart rate and body weight were monitored, blood was collected for the detection of some indexes like fibrinogen, total cholesterol and uric acid. Results demonstrated that blood pressure, whole blood viscosity, blood fibrinogen, total cholesterol and uric acid increased significantly both in the Tmin and Tr groups; low density lipoprotein/high density lipoprotein increased significantly only in Tr group; there was higher level of blood fibrinogen in the Tr group than the Tmin group; higher levels of creatine kinase-MB was found in both the Tmin and Tr groups. These results suggest that cold air may increase the cardiovascular risks in hypertensive subjects indirectly through its effects on the sympathetic nervous system and renin angiotensin system, blood pressure and atherosclerosis risk factors like blood viscosity and fibrinogen, lipids and uric acid in the blood.

## 1. Introduction

Epidemiological studies and clinical observations have demonstrated that cardiovascular diseases (CVDs) like myocardial infarction (MI) and coronary heart disease (CHD) events increase significantly in winter and cold weather. There was an 11% (95% confidence interval: 1.00–1.23, *p* = 0.04) increased risk of incident myocardial infarction (MI) during winter (November–January) compared with non-winter seasons in a sub-Arctic population [1]. The Lille-World Health Organization Monitoring Trends and Determinants in Cardiovascular Disease (MONICA) Project suggested that a 10 °C decrease of ambient temperature (Ta) was associated with a 13% increase in MI and coronary deaths event rates (*P < *0.0001) [2]. However, the mechanism involved is still not clear. Etiologically, the formation of atherosclerosis in the coronary artery is the cause of both MI and CHD [3]. Studies have reported the increase of blood viscosity, fibrinogen and cholesterol in subjects after cold stress and in winter [4,5,6,7]. As they are the promoters of atherosclerosis, the increased MI and CHD events in cold weather may be connected with them. Besides, cold stimulation as a stress, can increase sympathetic activity in the body [8]. The increased sympathetic activity may cause vasospasms [9] and aggravate CHD and induce MI. Therefore, the possible mechanism for the cold to increase MI and CHD events may be the combined effect of an activated sympathetic nervous system (SNS) and increased atherosclerosis risk factors, particularly in the subjects with CHD or MI. In addition, some other risk factors like uric acid may also play a role in that. Therefore, this study aimed to explore the effect of cold weather on cardiovascular system risk factors in spontaneously hypertensive rats (SHR). In order to simulate cold weather, we simulated an artificial cold air and used it as a cold stress for the animals. 

## 2. Materials and Methods

### 2.1. Cold Air Data and Cold Air Simulation

Zhangye city is located in northwest China and on the sole route for the northwest cold air of China. The cold air was monitored by the Lanzhou Central Meteorological Observatory and hourly Ta data was collected from the Zhangye Meteorological Observatory, from the 13th to the 15th of March of 2011 [10]. According to the Chinese cold air scaling criteria (GBT 20484–2006), this cold air belonged to the moderate cold air range. Among the analyzed 7 year cold air data of Zhangye City (2004–2010), this range occurred for about 103 times, thus accounting for almost 80% of all cold airs in the area; hence, it could be regarded as a frequently occurring cold air range in Zhangye City. The Tmin of the previous day was chosen as the starting Ta so as to scale the cold air more clearly. Details of this cold air are shown in Table 1. 

**Table 1:** Details of ther cold air.

Cold air rank	Tmin-s (°C)	Tr (°C)	Tmin-g (°C)	↓∆T48 (°C)	Tmin (°C)	↓∆Tmax (°C)	Rewarming duration (h)	Total duration (h)
**Moderate**	20	20	12.7	7.3	11.1	8.9	51	55

Tmin-s the starting minimum temperature of the cold air, Tr the rewarming temperature, Tmin-g the minimum temperature for cold air grading, Tmin the minimum temperature, ↓∆T48 = Tmin-s − Tmin-g, ↓∆Tmax = Tmin-s − Tmin.

The cold air was introduced using an intelligent climate simulator box (GDJS-500 L, Pulingte Co., Tianjin, China). It has a controllable temperature range of −20 °C–120 °C. Both shifted and constant temperature, atmospheric pressure and humidity can be obtained and controlled through the microcomputer control system in it as needed. Data on temperature, relative humidity, and atmospheric pressure variation can be automatically recorded every 10 s by an external computer. The animal chamber of this climate simulator box is an enclosed space of 80 cm × 80 cm × 80 cm, large enough to sustain experimental living conditions for the rats. The light in the chamber is controllable and provided a luminous flux similar to that of laboratory conditions. The oxygen concentration of the chamber can be sustained at levels similar to those in the laboratory through a fixed air vent. After repeated tests, the climate simulator was considered well suited to simulate temperature changes for this study.

### 2.2. Animals and Grouping

Male spontaneously hypertensive rats (SHR) weighing 210.9 g–246.5 g, aged 10 weeks and with SBP 162 mmHg–180 mmHg were obtained commercially from Vital River Laboratories, Beijing, China. Rats were randomly divided into Tmin, Tmin-c, Tr and Tr-c groups with six rats in each. Tmin group received the Ta dropping process exposure of cold air (from Tmin-s 20 °C to Tmin 11.1 °C), while Tr received the entire cold air process exposure. Tmin-s and Tr-c served as concurrent control groups of Tmin and Tr, respectively. 

### 2.3. Animal Housing for Control Period

Rats were kept in metal and plastic cages, receiving a circadian rhythm of 12 h/12 h light/dark (light lasted from 08:00 to 20:00), stable room temperature 20 ± (2) °C and relative humidity of 45 ± (5)% for 2 weeks as control housing. During that, sufficient standard normal rat chow and water were supplied for rats to intake *ad libitum*. Bedding was refreshed daily for every cage. This research was conducted in accordance with the Declaration of Helsinki and with the Guide for Care and Use of Laboratory Animals as adopted and promulgated by the United National Institutes of Health. All experimental protocols were approved by the Review Committee for the Use of Human or Animal Subjects of Nanjing University of Information Science and Technology.

### 2.4. Cold Air Exposure

After the control period, the Tmin and Tr groups were moved to the chamber of the climate simulator box, while the two control groups were still kept in the same environment as during the control period. Cold air was introduced via the climate simulator box with the pre-input cold air data. Rats received food and water *ad libitum*, daily refreshed beddings and a circadian rhythm of 12 h/12 h light/dark (light lasted from 08:00 to 20:00) during the exposure. The relative humidity was controlled at 45 ± (5)% throughout the cold air exposure. Cold exposure started at 05:00 in the early morning so as to simulate a normal Ta dropping process. In all, the temperature dropping process persisted for 51 h and the entire cold air lasted for 55 h until the initial temperature was regained (20 °C). Conditions of rats were observed for every six hours.

### 2.5. Monitoring of Blood Pressure, Heart Rate and Body Weight

Blood pressure (systolic blood pressure), heart rate and body weight of rats were monitored once a week during control period and before and after cold air exposure. Systolic blood pressure (SBP) and heart rate were monitored by the non-invasive tail-cuff method using an animal sphygmomanometer (BP-2006A, Softron, Beijing, China), which is widely used in many cold exposure experiments [11,12].

### 2.6. Plasma Collection

After abdominal anesthesia in rats with pentobarbital sodium (120 mg/kg ip), blood was collected in vacuum tubes with anticoagulant (liquaemin) through the abdominal aorta. Three milliliters of blood were used for whole blood viscosity (WBV) determination immediately; the rest blood was centrifuged at 3,000 rpm for 10 min to collect plasma and saved at −80 °C until assay. 

### 2.7. Assessment of Vasoconstrictors, Atherogenesis Risk Factors and Myocardium Injury Indicator

Before assay, the plasma kept at −80 °C was melted at 37 °C. Plasma level of norepinephrine (NE), epinephrine (EPI), endothin1 (ET1) and angiotensin II (ANG II), were determined with an ELISA kit (Uscnlife, Wuhan ElAab Science Co. Ltd., Wuhan, China) in a blinded manner following the manufacturer’s instructions; WBV was determined using a blood rheology system (LGR80, Steellex, Beijing, China) at shear rates of 10/s and 150/s. The Clauss method was used to measure the plasma concentration of fibrinogen (FG) (reagents produced by Siemens Healthcare Diagnostic Products Gmbh, Marburg, Germany) [13]. Methods of GPO-PAP, CAT, and CHOD-PAP determination were applied to obtain the triglyceride (TG), low density lipoprotein (LDL), and high density lipoprotein (HDL) levels and total cholesterol (TC) [14,15,16,17] (reagents produced by Sichuan Maker Biotechnology Co. Ltd., Chengdu, China). Plasma uric acid was determined with the uricase-peroxidase method [18]. Creatine kinase MB (CK-MB) mass concentration was assayed by selective inhibition method with the help of Beckman Coulter Synchron LX20 (reagent produced by Beckman Coulter Inc., Brea, CA, USA).

### 2.8. Statistical Analysis

Results were analyzed with SPSS13.0 for Windows and shown as mean (SE, standard error). Means between different treatment groups were compared by Independent-sample T test. A 95% confidence limit was employed to explore significance.

## 3. Results

### 3.1. SBP, Heart Rate and Body Weight

**Figure 1:**
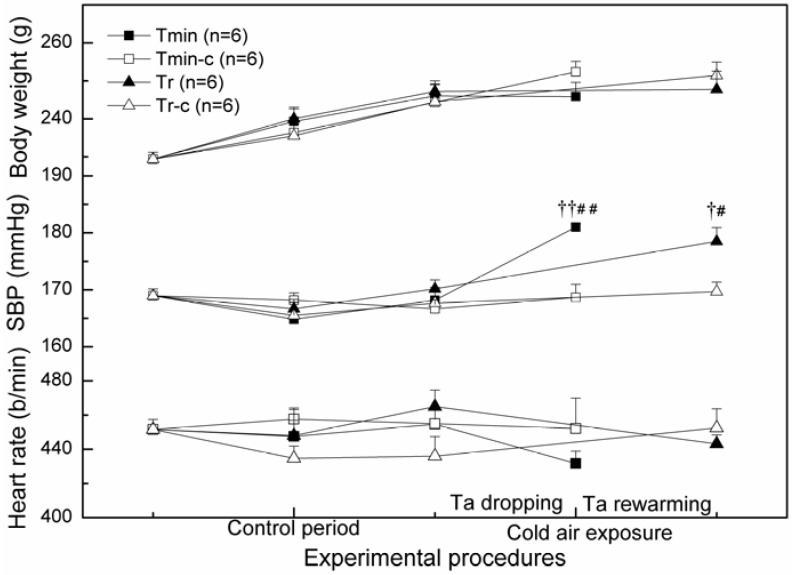
Variation of body weight, SBP and heart rate during control period and cold air treatment in different groups. SBP systolic blood pressure; Ta ambient temperature. Compared with control group ^† ^*P < *0.05, ^†† ^*P < *0.01; Compared with precious value ^# ^*P < *0.05, ^## ^*P < *0.01.

Figure 1 shows the variations of body weight, SBP and heart rate of rats throughout the control period and cold air exposure. There was significant elevation in SBP before and after cold air exposure both for Tmin group and Tr group. Compared to their control groups, their SBP were also significantly higher (*P < *0.01, *P < *0.05). However, there was no significant difference with regard to body weight and heart rate between control group and experimental group as well as before and after cold exposure in all groups (*P > *0.05). No significant difference was found between Tmin and Tr group in either SBP, heart rate or body weight (*P > *0.05).

### 3.2. Vasoconstrictors—EPI, NE, ANGII and ET-1

**Figure 2:**
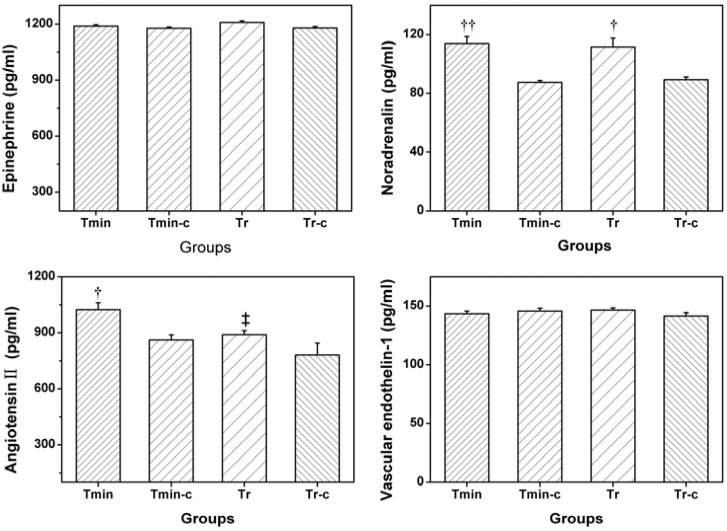
Norepinephrine, epinephrine, endothin l and angiotensin II in different groups (n = 6). Compared with control group ^† ^*P < *0.05, ^†† ^*P < *0.01; compared with Tmin group ^‡ ^*P < *0.05.

The concentration of plasma EPI, NE, ANGII and ET-1 for all groups are represented in Figure 2. There was no significant difference in plasma NE and ET-1 between any two groups of the four groups (*P > *0.05). Significantly higher level of plasma NE occurred in both the Tmin and Tr groups when compared with their control groups (*P < *0.01, *P < *0.05). No significant differences were found between the Tmin and Tr groups (*P > *0.05). With regard to plasma ANGII, a markedly higher level was measured in the Tmin group, higher than the Tr group and its group (*P < *0.01, *P < *0.05). No obvious difference was found between the Tr and Trc group as well as between the Tmin-c group and Trc group (*P > *0.05).

### 3.3. CK-MB and Atherogenesis Risk Factors—WBV, Blood Lipids, FG and Uric Acid (Table 2)

Both in the group of Tmin and Tr, concentration of WBV (*P < *0.01, *P < *0.05) and plasma FG (*P < *0.01, *P < *0.01), TC (*P < *0.01, *P < *0.05), LDL (*P < *0.05, *P < *0.05) and uric acid (*P < *0.01, *P < *0.01) were significantly higher than their control groups, while the plasma TG was significantly lower (*P < *0.05, *P < *0.05). 

**Table 2:** CK-MB and atherosclerosis risk factors-WBV, FG, blood lipids and uric acid in different groups.

Atherosclerosis risk factors		Tmin-c (n = 6)	Tmin (n = 6)	Tr-c (n = 6)	Tr (n = 6)
Whole blood	10/s	11.10 (0.58)	17.31 (0.49) ^††^	12.36 (0.76)	15.97 (2.21) ^†^
Viscosity (mPa.s)	150/s	4.29 (0.10)	5.73 (0.14) ^††^	4.53 (0.13)	5.25 (0.39) ^†^
Fibrinogen (g/L)		1.92 (0.04)	2.06 (0.01)^ ††^	2.00 (0.04)	2.30 (0.06) ^††,‡‡^
Blood lipids (mmol/L)	TC	0.89 (0.01)	1.14 (0.04) ^††^	0.93 (0.02)	1.08 (0.04) ^†^
	TG	0.38 (0.04)	0.27 (0.02) ^†^	0.39 (0.04)	0.28 (0.01) ^†^
	HDL	0.5 (0.01)	0.62 (0.01) ^††^	0.56 (0.02)	0.55 (0.02) ^‡^
	LDL	0.48 (0.03)	0.65 (0.04) ^†^	0.41 (0.02)	0.73 (0.10) ^†^
LDL/HDL		0.97 (0.07)	1.06 (0.06)	0.74 (0.01)	1.33 (0.22) ^††^
Uric acid (umol/L)		71.5 (5.67)	173.67 (17.78) ^††^	71.17 (7.01)	170.67 (18.62) ^††^
CK-MB (U/L)		236.17 (20.98)	362.67 (29.37) ^††^	214.5 (9.25)	378.67 (25.88)^ ††^

TC total cholesterol, TG triglyceride, HDL high density lipoprotein, LDL low density lipoprotein. Compared with control group ^† ^*P < *0.05, ^†† ^*P < *0.01; compared with Tmin group ^‡ ^*P < *0.05, ^‡‡ ^*P < *0.01.

There was also obviously higher level of HDL in the Tmin group (*P < *0.05) than in its control group but not in the Tr group (*P > *0.05), contrary to the higher value of LDL/HDL in the Tr group (*P < *0.05) than its control group but not in the Tmin group (*P > *0.05). Between the Tmin group and Tr group, significant differences were only seen in plasma FG and LDL (*P < *0.01, *P < *0.05). In the CK-Mb result, both the Tmin and Tr groups showed higher levels than their control groups (*P* < 0.01, *P* < 0.01). No significant differences existed between the Tmin group and Tr group (*P* > 0.05).

## 4. Discussion

In this study, cold air increased the level of atherosclerosis risk factors both in the Tmin and Tr groups, which indicates that CVDs risk may increase in SHR rats during cold air exposure. Atherosclerosis risk factors like higher blood pressure, WBV, plasma FG, TC and uric acid were all higher in the treatment groups than control groups. Blood pressure elevation increases the heart load and cardiac oxygen exhaustion [19,20], which may ultimately contribute to the occurrence of myocardial infarction. Besides, the elevated blood pressure interferes with the integrity of endothelial cells and cause endothelial activation or dysfunction to form atherosclerosis in the vasculature [21,22]. The increase in WBV usually indicates the increase in some coagulation factors, such as red cells, FG, blood lipids, platelet, and haemoglobin [7,23], which are essential factors for the formation of atherosclerosis. FG, as an indispensable coagulation factor, can directly participate in the process of atherosclerosis by binding fibrin and its degradation products—FDP—to induce pro-inflammatory responses [24]. TC and LDL are closely related to the induction of atherosclerosis by inducing adhesion molecules and vascular endothelium dysfunction manifestations [25,26]. Increased uric acid in blood is thought to play a role in the proliferation of vascular smoothness and stimulation of inflammatory pathways to induce endothelial dysfunction [27,28,29], which may in turn promote atherogenesis. These factors increased in rats after cold air exposure and may facilitate the atherosclerosis formation and aggravate any preexisting atherosclerosis, especially in coronary arteries. The literature reports that cold exposure reduced the cardiac antioxidant capacity and contributed to cardiac fibrosis and other abnormalities [30]. Therefore, an increase of an oxidant like LDL as well as uric acid by cold air exposure may promote cardiac abnormalities. In addition, SHR rats are characterized not only by higher blood pressure but also exaggerated cardiovascular and sympathetic responses to the stimuli like cold [31,32]. Their innate higher blood pressure may have already induced atherosclerosis in the coronary artery by endothelial activation or dysfunction [33]. Combined with the vasospasms caused by increased vasoconstrictors like NE and ANGII during cold air exposure, SHR may be more likely to be affected by cold air, especially in the cardiovascular system. Therefore, the long-term affected coronary artery by higher blood pressure and the increased SNS and RAS activities as well as above atherogenesis promoters after cold air exposure may comprehensively contribute to the rise of cardiovascular risk in cold air-treated SHR rats. 

Epidemiological studies report that there is a lag effect of cold in CVD events, including MI and CHD [34]. The higher level of FG and LDL/HDL in the Tr group may further indicate the higher MI and CHD risk after the Ta dropping process. The LDL/HDL ratio is an excellent predictor of CHD risk, being more accurate than LDL or HDL alone [35]. The proper explanation may be that the atherogenesis is a slow process and the increased atherogenesis risk factors need time to recover, therefore, MI and CHD event peaks may occur after cold exposure. 

Epidemiological studies have reported that there were higher CVDs risks during cold exposure. In the Dutch population from 1979–1997, the estimated average excess mortality during cold spells was 12.8% or 46.6 deaths/day, largely attributable to the increase in cardiovascular mortality [36]. In this study, the increased SNS and rennin-angiotensin system (RAS) activity, SBP, WBV, plasma FG, TC and uric acid during cold air exposure might contribute to the higher cardiovascular risk in the treatment groups. Therefore, this study provide a directly explanation for the increased CVDs risk during cold weather in the cardiac disease aspect. The elevated blood pressure is probably induced by the increased NE and ANGII during cold air exposure, as both the blockers of beta-adrenergic receptors and ANGII have been reported to reduce the elevated blood pressure during cold exposure [37,38]. Therefore, the blood pressure elevation can be prevented by the administration of these antihypertensives. Besides, as other atherogenesis risk factors increase may be caused by the blood concentration during cold exposure [4], more water drinking should be suggested. In this way, this study provides signs that the reduction of these risk factors may reduce the MI and CHD risk in cold weather. However, further studies are needed to identify whether this could work. In addition, concerning the increased atherogenesis risk factors in the hypertensive subjects, the hypertensive population should pay more attention to the cold weather effect, as epidemiological studies have already found the higher CVDs risk in the CVDs population [39,40].

This study applied an artificial cold air process as cold stress, which better represents the cold environment in Nature. Naturally, cold air involves a slow process of Ta fluctuation (including Ta dropping and Ta rewarming), which is substantially different from the cold in many other studies. Many cold studies exposed subjects to a constant lower Ta from a warm environment within minutes, such as from 22 °C to 4 °C in 8 min [12]. The sharply dropped Ta induced an apparent blood pressure elevation through the activation of SNS and RAS [8]. Similarly, cold air with a slow Ta fluctuation also induced blood pressure elevation through the activation of SNS and RAS, which further adds to the knowledge of cold-induced blood pressure elevation. Other studies have demonstrated the increase of atherogenesis risk factors like blood pressure, blood viscosity, blood FG and cholesterol, but they have not evaluated the CVDs risk by some indicators like blood CK-MB [4,5,6,7]. The results of our study may be used to explain the phenomenon of increased CVDs risk in cold weather. 

Notwithstanding the many strengths of this study, its many limits should also be discussed. During the entire experiment, the core temperature (Tc) of SHR was not monitored. Therefore, the variation of Tc in SHR rats under Ta fluctuation was not understood, even if rats are able to maintain their core temperatures constant during cold exposure to 5 ± (2) °C [41,42]. In the experimental design, the time points for the atherogenesis risk factors and hormone monitoring were limited to Tmin and Tr, which limited our horizon to their levels at only Tmin and Tr without a panoramic understanding of their variation during the whole Ta change of cold air. Besides, the Ta rewarming process was few hours without sustainment, which may not be convincing enough to explain the lagged CVDs events peak in several days in cold weather [34]. Hence, our next experiment will focus on the atherogenesis risk factors and hormone variation along with the Ta variation of cold air and design longer intervals after cold air exposure to try to explain the lagged CVDs events peak. What is more, this study may only explain the increased CVDs risk among subjects who cannot take effective warm preservation measures and have to experience the cold air. Studies over the connection between atherogenesis risk factors and CVDs risk in a sudden cold exposure are still necessary to explain the increased CVDs risk in cold weather. Even though this study checked some risk factors of CVDs in cold air exposure, the mechanism by which they cause cardiac abnormalities is still unclear. Therefore, more in-deepth studies are needed to explore the specific role of these CVDs risk factor and the specific mechanism in causing CVDs during cold exposure.

In conclusion, cold air may increase the cardiovascular risks in hypertensive subjects indirectly through its effects over sympathetic nervous system and rennin-angiotensin system, blood pressure and atherosclerosis risk factors like blood viscosity and fibrinogen, lipids and uric acid in the blood.
